# Stochastic motion and transcriptional dynamics of distal enhancer–promoter pairs on a compacted chromosome

**DOI:** 10.1101/2023.01.18.524527

**Published:** 2023-01-20

**Authors:** David B. Brückner, Hongtao Chen, Lev Barinov, Benjamin Zoller, Thomas Gregor

**Affiliations:** 1 Institute of Science and Technology, Am Campus 1, 3400 Klosterneuburg, Austria; 2 Lewis-Sigler Institute for Integrative Genomics, Princeton University, Princeton, NJ, USA; 3 Joseph Henry Laboratories of Physics, Princeton University, Princeton, NJ, USA; 4 Department of Developmental and Stem Cell Biology, CNRS UMR3738, Institut Pasteur, Paris, France

## Abstract

Chromosomes in the eukaryotic nucleus are highly compacted, with a crowded polymer organization of fractal dimension three. However, for many functional processes, including transcription initiation, the 3D pair-wise motion of distal chromosomal elements, such as enhancers and promoters, is essential and necessitates dynamic fluidity. Therefore the interplay of chromosome organization and dynamics is crucial for gene regulation. Here, we use a live imaging assay to simultaneously measure the positions of pairs of enhancers and promoters and their transcriptional output in the developing fly embryo while systematically varying the genomic separation between these two DNA elements. Our analysis reveals a combination of a compact globular organization with fast subdiffusive dynamics. These combined features cause an anomalous scaling of polymer relaxation times with genomic separation and lead to long-ranged correlations compared to existing polymer models. This scaling implies that enhancer–promoter encounter times are much less dependent on genomic distance than predicted by existing polymer models, with potentially significant consequences for eukaryotic gene expression.

Living systems are built based on information encoded in chromosomes confined in each cell’s nucleus. These meter-long DNA polymers must be highly compacted to fit into the micron-sized structure [1, 2]. At the same time, for cells to function, chromosome organization must allow the information content to be accessed and read out through transcription [3, 4]. Often transcription can only occur through the spatial interaction of DNA loci, such as enhancers and promoters. They find each other dynamically and remain in physical proximity [5–8]. While the distances over which many enhancers function in higher eukaryotes can be up to mega-base pairs in genomic separation [9–12], it is unknown how these elements come into proximity, what their typical distance is in 3D space, and how they explore this space dynamically in the process. Specifically, it remains unclear how the real-time physical motion of such coupled pairs of DNA loci determines transcriptional encounters and how this depends on their genomic separation.

Over the past decade, the advent of chromosome capture and imaging methods [13] has given key insights into the 3D spatial organization of chromosomes, with the discovery of structural features such as topologically associated domains (TADs) [14–17], phase-separated nuclear condensates [18–20], and larger-scale compartments [21, 22]. At larger length scales, across tens to hundreds of TADs, chromosome organization has been suggested to be highly compacted in a crumpled chain configuration (often referred to as crumpled or fractal globule) [22–26], a long-lived polymer state with fractal dimension three.

At the same time, live-imaging experiments have given insight into the real-time dynamics of individual DNA loci [27–32]. These dynamics exhibit subdiffusion in a broad range of systems and follow well a characterization by the simple Rouse polymer model [27, 28, 31, 32]. It describes polymer dynamics as a collection of diffusing beads linked by linear elastic springs [33]. However, the Rouse polymer configurations exhibit ideal chain statistics, leading to a loosely packed polymer configuration with fractal dimension two that contrasts the compacted architecture of the crumpled chain model.

Based on these findings, our current understanding of chromosome organization and dynamics suggests two contradictory polymer physics frameworks, which inevitably blurs any implications for parameters of enhancer-promoter interactions and transcriptional activity. In addition to simulation-based models for chromosome dynamics [25, 29, 34–39], a promising technique to address this gap is scaling approaches that combine fractal organization and sub-diffusive dynamics [40–42]. Indeed, recent theoretical work provides scaling predictions for the two-body dynamics of coupled chromosomal loci [36, 41, 42], but these have never been tested experimentally. Thus far, experimental data sets have given insight into either static organization [14–17, 22, 25], dynamic properties of chromosomes [27–29, 31, 32], or transcription [8, 30, 43, 44], but rarely at the same time. For instance, previous live measurements of locus pairs occurred at fixed genomic separation in transcriptionally silent loci [31, 32]. To investigate how 3D spatial organization and dynamic locus motion control the time scales of enhancer–promoter encounters and thus transcriptional activation, we require an approach to simultaneously monitor the movement of DNA locus pairs and transcription across a series of genomic separations *in vivo*.

Here, we address this problem by live imaging the joint dynamics of two cis-regulatory DNA elements, an enhancer, and a promoter, while simultaneously monitoring the transcriptional output resulting from their dynamic encounters in developing fly embryos. We systematically vary the genomic separation between the enhancer–promoter pair spanning many TADs. Stochastic real-time trajectories of the 3D motion of the two loci show a dynamic search process, with physical proximity required for successful transcription and a power-law scaling of transcription probability with genomic separation. While typical 3D distances between the locus pair follow a compact packing consistent with the crumpled chain model, the dynamic properties exhibit fast diffusion, albeit with a diffusion coefficient that increases with genomic separation, a seemingly optimal combination of static and dynamic features of the system. These features give rise to an anomalous scaling of enhancer–promoter encounter times and long-range correlations in the dynamics of the equilibrated polymer. This suggests that the enhancer–promoter search process is much less dependent on genomic separation than expected based on existing polymer models.

## Live imaging of chromosome dynamics and transcription.

**Figure 1.**
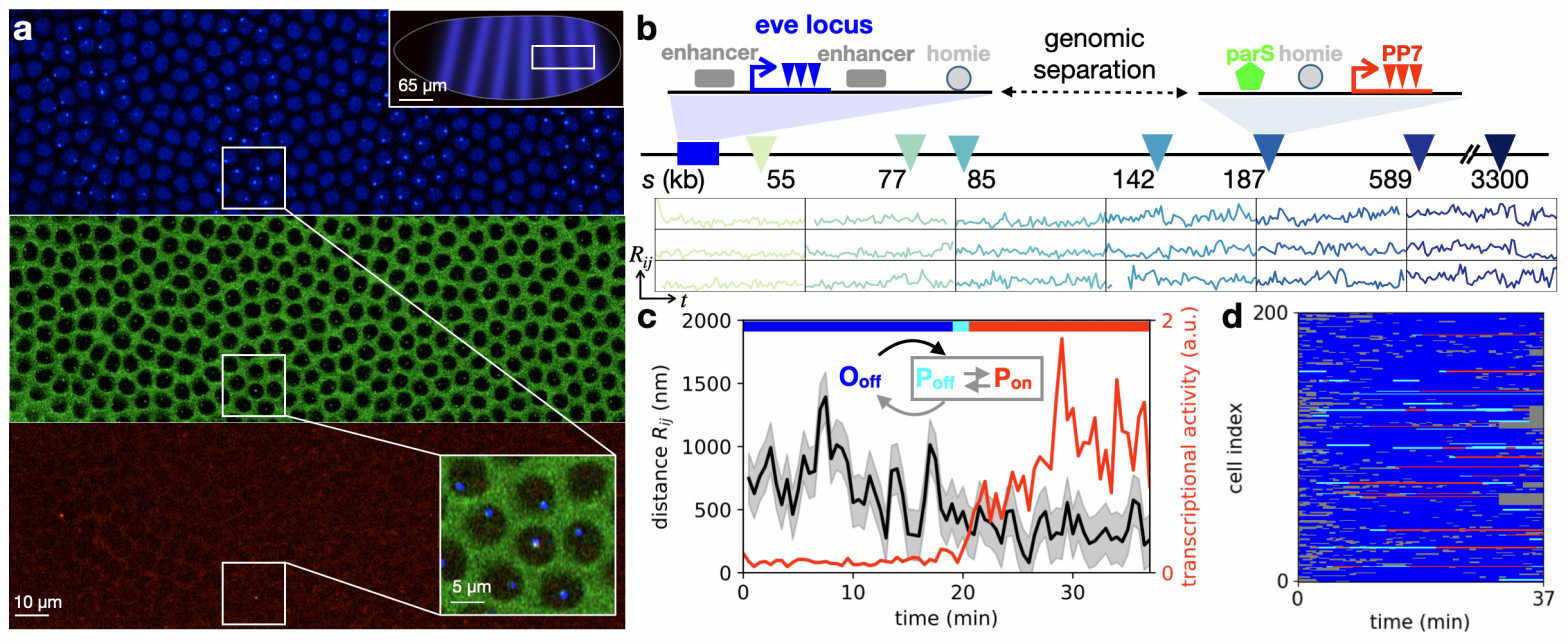
Simultaneous tracking of DNA loci and transcriptional activity in living embryos. **a**, Typical surface view of a representative fly embryo, displaying fluorescent foci for MS2, PP7, and parS in the corresponding blue (top), green (center), and red (bottom) channels. Top inset shows schematic with image location in the embryo; bottom inset shows a close-up. **b**, Top: schematic of the gene cassettes used for three-color imaging. The endogenous *eve* locus (left) is tagged with MS2 stem-loops that are labeled via blue fluorescence. A reporter with an *eve* promoter driving PP7 transcription (labeled via red fluorescence) is integrated at a genomic separation *s* from the *eve* locus on the 2^nd^ chromosome in the *Drosophila* genome. It includes a homie insulator sequence allowing loop formation through homie–homie pairing, and a parS sequence that is permanently labeled with green fluorescence. Seven such constructs were generated with varying genomic separation *s* (triangles). Bottom: sample inter-locus distance trajectories *R*(*t*) for six genomic separations, with standardized y-axis limits (0, 2 μm) and x-axis limits (0, 30 min), obtained following nucleus and locus segmentation, tracking, chromatic aberration and motion correction (Supplementary Section 2.1, 2.2). Sampling time interval is 28 s. **c**, Trajectories of inter-locus distance *R* and transcriptional activity, with inferred topological states shown by colored top bar (Supplementary Section 2.3). Inset: Schematic of the three topological states. **d**, 200 examples of state trajectories sampled from a total set of *N* = 579 trajectories acquired in *n* = 30 embryos (genomic separation *s* = 142kb).

To simultaneously monitor the coupled motion of enhancer–promoter pairs and transcription across multiple genomic separations, we generated fly lines, in which a reporter gene is introduced at various genomic locations from the well-studied *Drosophila even-skipped (eve)* locus (Fig. 1b). The locations of both the endogenous *eve* enhancers and the promoter of the reporter gene, as well as the transcriptional activity of the reporter gene are measured together using a three-color imaging system (Fig. 1a, Supplementary Section S1) [8]. To facilitate transcription, the reporter cassette additionally contains the insulator element *homie*, which allows stable loop formation with the endogenous *homie* element in the *eve* locus. As demonstrated previously [8], this system exhibits three topological states (Fig. 1c): an open configuration O_off_ where the *homie* elements are not bound to each other, and two paired configurations P_off_ and P_on_, where a loop is formed with either inactive or active transcription, respectively. We build seven of such reporter constructs, with genomic separations *s* varying over close to two orders of magnitude from 55kb to 3300kb, comparable to the distances over which many enhancers function in higher eukaryotes [9–12]. Importantly, these genomic length-scales span across multiple TADs in the *Drosophila* genome, where median TAD sizes are typically 90 kb [45] (here 18 kb for the *eve* locus [46]).

## Inter-locus distance scaling suggests crumpled chain organization.

**Figure 2.**
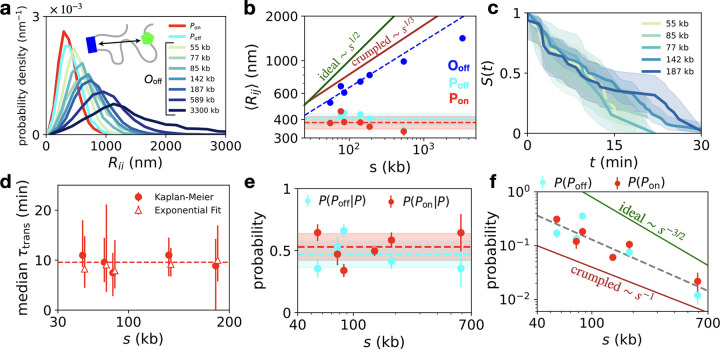
Scaling of enhancer–promoter distances and transcriptional activity across genomic separations. **a**, Probability distributions of the inter-locus distances *R*. Distributions are separated by state, with paired states pooled across genomic separations, and individual distributions shown for the open state. **b**, Average inter-locus distances ⟨*R⟩* for each of the three transcriptional states. Blue dashed line indicates a linear best fit to the O_off_ data for the range of genomic separations 55–187 kb, with exponent 1*/d* = 0.31±0.07. Dashed cyan and red lines are average values of the interlocus distances of the P_off_ and P_on_ states, respectively, with shaded areas indicating error of the mean. Solid dark green and red lines indicate predictions for ideal and crumpled polymers, respectively. **c**, Survival curves *S*(*t*) of the transcriptionally active state P_on_, giving the probability that transcription remains active after time *t*. We use the Kaplan-Meier estimator for *S*(*t*) which accounts for censoring, which occurs if the trajectory begins or ends in the transcriptionally active state. Shades show 95% confidence intervals [31] (Supplementary Section S2.5). **d**, Median lifetime of the transcriptionally active state P_on_ as a function of genomic separation, using the Kaplan-Meier estimator (dots) and a maximum-likelihood estimator assuming exponential decay of the survival curves (triangles; Supplementary Section S2.5). **e**, Probability of the paired on and off states conditioned on the system being in one of these two paired configurations. **f**, Overall probability of the paired configurations P_off_ and P_on_ as a function of genomic separation. Dashed line indicates best fit with exponent 1.16 ± 0.18. Green and dark red lines indicate predicted exponents for the contact probabilities of the ideal and crumpled chain polymer models.

**Figure 3.**
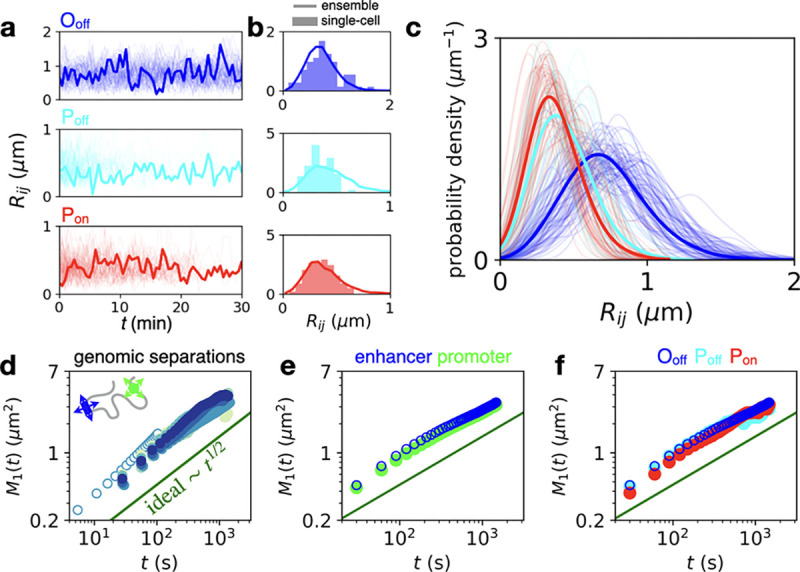
Dynamics of enhancer–promoter search and single-locus fluctuations. **a**, Single-cell inter-locus distance trajectories for the three topological states (*s* = 142 kb). For each state, 80 trajectories are shown, with one sample trajectory highlighted in bold. **b**, Distance distributions of the highlighted trajectory in panel c (bar histogram) compared to the ensemble distribution obtained by averaging over all cells (line). **c**, Single-cell interlocus distance distributions of all trajectories in panel c (thin lines) for the three states compared to ensemble distributions in bold (bold lines) (*s* = 142 kb). Distributions are smoothed using Gaussian kernel density estimation with a width of 100 nm. Only trajectories with at least 10 time-points are included to ensure sufficient statistics for comparison. **d**, Single-locus MSDs for all genomic separations (color code corresponds to Fig. 2a). Open data points correspond a shorter imaging time-interval Δ*t* = 5.4s (*s*=142kb). **e**, Single-locus MSDs comparing enhancer (blue) and promoter (green) fluctuations (*s* = 142 kb). **f**, Single-locus MSDs comparing fluctuations in the three states (*s* = 142 kb).

To determine the instantaneous topological and transcriptional state of the system, we adopt an inference approach using a Hidden Markov Model based on the time-series of interlocus distance and transcriptional activity (Supplementary Section S2.3). We assign one of these states to each measured configuration, including the hidden P_off_ state (Fig. 1d). A key question is then how the 3D enhancer–promoter distances *R* in the open configuration O_off_ vary with the linear genomic separation *s*. These distances exhibit broad distributions, which shift systematically with larger separation (Fig. 2a). From a polymer physics perspective, the mean distance ⟨*R*⟩ is expected to scale as *s*^1*/d*^, where *d* is the fractal dimension: while an ideal chain polymer, as predicted by the simple Rouse model, has fractal dimension *d* = 2, the compact crumpled chain organization has dimension *d* = 3 [24, 47]. In our experiments, we observe a scaling exponent of 1*/d* = 0.31±0.07 for genomic separations up to *s* = 589 kb, consistent with the crumpled chain model (Fig. 2b). The smaller than expected average distance (⟨*R*⟩~1.5 *μ*m) observed for the largest separation (*s* = 3300 kb) is most likely affected by the average folding of the chromosome [48]. As anticipated, we find that the distances of the paired configurations do not scale with genomic separation, and exhibit distances of 350−400nm (Fig. 3b), consistent with previous measurements of distances within the *eve*-locus [8, 49]. Together, these results reveal a compact crumpled chain architecture of chromosome configurations in a range of genomic separations consistent with Hi-C experiments in *Drosophila* [17].

## Transcriptional activity scales with genomic separation.

From the latent state trajectories revealed by our inference approach, we estimate the survival curves of the transcriptionally active state, allowing calculation of the transcription lifetimes (Fig. 2c). We find a median lifetime independent of genomic separation close to 10 min (Fig. 2d). This corresponds to about 3–5 independent rounds of transcription on average, given the typical promoter switching correlation time of the system [51]. Similarly, the relative proportion of transcriptionally active states within the paired subpopulation is insensitive to genomic separation (Fig. 2e).

In contrast, the overall probability of observing either of the paired configurations strongly depends on genomic separation, and exhibits a power-law scaling *P*(*s*) ~ *s*^−*f*^, with *f* = 1.13 ± 0.16 (Fig. 2f). Since transcriptional lifetimes are independent of distance, the scaling of *P*(*s*) is likely dominated by the search of the two loci to come into contact. Importantly, different polymer models make distinct predictions of the scaling of contact probabilities [22, 24, 52]: for ideal chain, *f* = 3*/*2, while a crumpled chain exhibits *f* = 1, which is consistent with the scaling we observe. Together, these results suggest that the transition from the open to the paired configuration is a key limiting step in transcriptional activation of distal enhancer–promoter pairs which depends on their genomic separation.

## DNA loci exhibit a subdiffusive search process.

To understand the time-scales of the DNA locus motion, we consider the real-time dynamics of the blue and green labelled DNA loci. Interestingly, we find that single trajectories in each of the topological states sample the entire ensemble-averaged distance distribution (Fig. 3a–c, Supplementary Fig. S3), suggesting ergodicity of the system. Thus, rather than existing in constrained configurations as observed in other genomic contexts [29], this observation supports the picture of a dynamic search process.

The motion of each individual DNA locus is quantified by the single-locus MSD *M*_1_(*t*) = ⟨(**x**_*i*_(*t*_0_ + *t*) − **x**_*i*_(*t*_0_))^2^⟩_*t*0_ = Γ*t*^*β*^, where **x**_*i*_(*t*) is the 3D position of the locus, Γ the diffusivity, and *β* the dynamic exponent. Based on a generalized Rouse approach, the dynamic properties of the polymer can be related to the static organization that is defined by the fractal dimension via *β* = 2*/*(2 + *d*) [40, 42, 53]. The key assumption of this approach is that friction acts locally on every monomer of the polymer. Thus, while the ideal chain Rouse model predicts *β* = 1*/*2, we expect *β* = 2*/*5 for a crumpled polymer. Interestingly, we measure a scaling exponent *β* = 0.58±0.09 across genomic separations, for both enhancer and promoter loci, close to the prediction of the ideal chain model, and consistent with previous works [28, 31, 32] (Fig. 3d,e). Interestingly, our data further indicate that the single-locus dynamics are not affected by transcriptional activity [43], as they are consistent across the three topological states (Fig. 3f).

**Figure 4.**
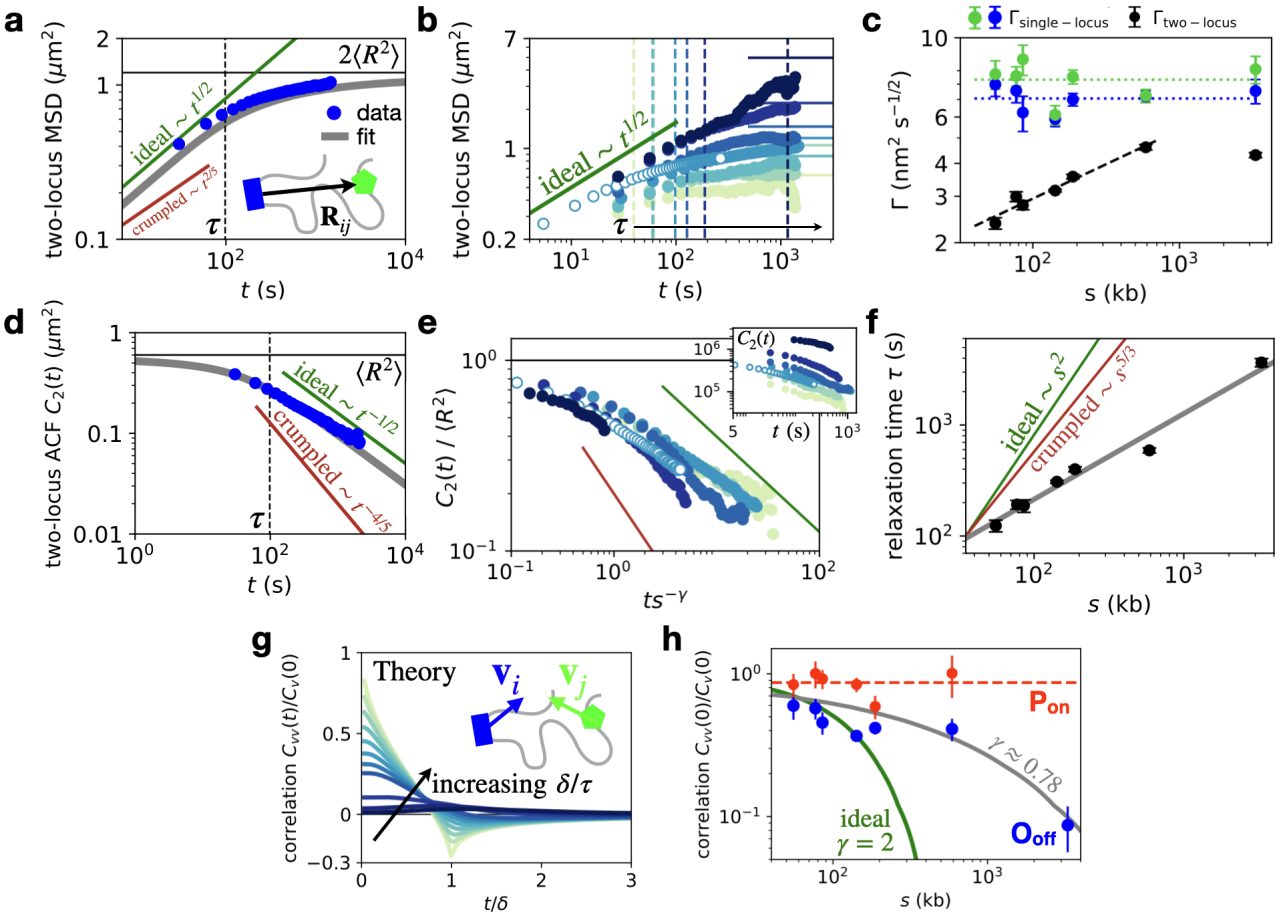
Joint dynamics of enhancer-promoter pairs. **a**, Rouse theory prediction of the two-locus MSD *M*_2_(*t*) = 2Γ*t*^1*/*2^(1 − *e*^−*τ/t*^) + 2*J* erfc[(*τ/t*)^1*/*2^] [31] (grey line), using best fit values Γ,*J*, *β* = 1*/*2, and $\tau =\frac{1}{\pi }{\left(J/\Gamma \right)}^{2}$; compared to experiment (*s* = 142kb). Green and red lines give expected scaling *t*^*β*^ for *t* ≪ *τ* for the generalized Rouse model for ideal and crumpled chains (Supplementary Section S4). **b**, All experimental two-locus MSDs with relaxation times (dashed lines) and expected asymptotes 2⟨*R*^2^⟩ (solid lines; color code corresponds to Fig. 2a). **c**, Scaling of the diffusion coefficients Γ from two-locus MSD fits (black dots), compared to single-locus diffusion coefficients obtained from single-locus MSDs (Fig. 3f–h) for enhancer (blue) and promoter (green). Dashed line: best fit to two-locus diffusivity with exponent 0.27 ± 0.03 (*s* = 55 − 589 kb) (Supplementary Section S5.1); dotted lines: average values of single locus diffusivities. **d**, Two-locus autocorrelation function (ACF) *C*_2_(*t*) = ⟨**R**(*t*_0_) · **R**(*t*_0_ + *t*)⟩_*t*0_ = ⟨*R*^2^⟩ − *M*_2_(*t*)*/*2 (grey) compared to data (*s*=142kb). Green and red curves indicate the power-law exponent *λ* = 2(1 − *d*)*/*(2 + *d*) of the correlation function *C*_2_(*t*) ~ *t*^*λ*^ for ideal and crumpled chains for *t* ≫ *τ*, respectively [42]. **e**, Collapsed correlations C_2_ ~ *C*_2_(*ts*^−*γ*^)*/*⟨*R*^2^⟩ with *γ* = 0.78. *Inset:* raw correlations *C*_2_(*t*) for varying genomic separation. Open data points correspond to data obtained with a higher sampling rate. **f**, Scaling of inferred relaxation times compared to predicted ideal and crumpled chain exponents. Grey line: best fit with exponent *γ* = 0.78±0.07. **g**, Predicted velocity cross-correlation functions $C_{\text{vv}}^{\left(\delta \right)}\left(t\right)=\langle v_{i}^{\left(\delta \right)}\left(t_{0}\right)\cdot v_{i}^{\left(\delta \right)}\left(t_{0}+t\right)\rangle t_{0}$ for increasing values of the dimensionless ratio *δ/τ* [50]. Velocities are calculated on a time-interval *δ* as **v**^(*δ*)^(*t*) = (**x**(*t*+*δ*)−**x**(*t*))*/δ*. **h**, Scaling of the zero-time velocity cross-correlation intercept normalized by the zero-time auto-correlation, $C_{\text{vv}}^{\left(\delta \right)}\left(0\right)/C_{\text{v}}^{\left(\delta \right)}\left(0\right)$, for the O_off_ (blue) and P_on_ (red) states; *δ* = 300s. Green line: prediction based on ideal chain Rouse scaling of the relaxation times (*γ* = 2) with an intercept determined based on the 55 kb data-point; grey line: parameter-free prediction using the inferred anomalous relaxation time scaling (*γ* ≈ 0.78) (Supplementary Section S6); dashed red line: average correlation in the P_on_ state.

We next turn to the joint dynamics of two coupled chromosomal loci. These dynamics are determined by the interplay of organization, single-locus dynamics, and the relaxation of the polymer. To quantify these dynamics, we analyze the statistics of the 3D distance vector **R**(*t*) via the two-locus MSD *M*_2_(*t*) = ⟨(**R**(*t*_0_ + *t*) − **R**(*t*_0_))^2^⟩_*t*0_ [31]. Here, the generalized Rouse approach provides a useful benchmark as it includes only the simplest mechanical communication through linear elasticity along the backbone of the polymer. In this model, *M*_2_(*t*) exhibits two distinct regimes: independent diffusion *M*_2_(*t* ≪ *τ*) = 2Γ*t*^*β*^ at short times below the relaxation time-scale *τ*, followed by a cross-over to a plateau, which is given by the size of the equilibrated chain *M*_2_(*t* ≫ *τ*) = 2⟨*R*^2^⟩ (Fig. 4a). Consistent with the observed single-locus dynamics, we find that the experimental two-locus MSDs for the various genomic separations are well fitted by the Rouse prediction (Fig. 4a,b). The equilibration of the system can be further quantified by the two-locus auto-correlation, which similarly exhibits a decay closer to the ideal chain exponent (Fig. 4d). Taken together, we find enhancer–promoter motion to exhibit an ergodic search process with dynamic diffusion exponents that are in line with the ideal chain expectations.

## Relative DNA locus motion reveals scale-dependent diffusivity.

Interestingly, the observed two-locus dynamics reveal that our system achieves much larger displacements in the same time than in mammalian stem cells [31, 32], suggesting comparatively fast chromosome dynamics (Fig. 4b). To extract the two-locus diffusion coefficients directly from the trajectories, we perform a Bayesian fitting of the two-locus MSD using the full distance trajectories [31] (Supplementary Section S5). For homogeneous polymer models, such as ideal or crumpled chains, one would expect the two-locus diffusivity to be independent of genomic separation, and to match the single-locus diffusivity obtained from the single-locus MSDs. Remarkably, however, we find that the two-locus diffusion coefficient increases with genomic separation up to 589 kb, with an approximate scaling Γ(*s*) ~ *s*^0.27±0.03^ (Fig. 4d). In contrast, we find that the single locus diffusion remains constant (Fig. 3d, 4d).

## Inter-locus relaxation times exhibit an anomalous scaling with genomic separation.

Having established the static and dynamic properties of the system, we now ask about the consequences of these features for the time-scales of the two-locus search process. Thus, we seek to infer the relaxation time scaling with genomic separation *τ* ~ *s*^*γ*^, which quantifies how fast stress propagates through the system, and sets the time taken for the two loci to come into proximity. The relaxation time is determined by the interplay of chromosome dynamics and organization: it is directly related to the time taken by the two loci to diffuse (dynamics) over their typical distance of separation (organization): Γ(*s*)*τ*^*β*^ ~ *s*^2*/d*^. This yields *γ* = 2 for the ideal chain and *γ* = 5*/*3 for the crumpled chain. Remarkably, due to the combination of static and dynamic exponents in our system, as well as the scale-dependent diffusivity, we find an anomalous relaxation time scaling with an exponent *γ* = 0.78 ± 0.06 (Fig. 4f). This result is further confirmed by a data collapse of the two-locus auto-correlation functions (Fig. 4e, Supplementary Section S5.2). Importantly, our inference results on the joint DNA locus motion are independent of the separation into topological states, as the paired states only represent a small subset of the trajectories, and the exponents inferred from a pooled data set including all states are consistent with those of the open state only (Supplementary Section S5.3).

In sum, we demonstrate that the relaxation time, which sets the time-scale of enhancer–promoter encounters, is much less dependent on genomic separation than predicted by existing polymer models. Indeed, for an ideal Rouse polymer, the relaxation time or our largest genomic separation (3.3 Mb) would be ~ 3600 times longer than for the shortest 55 kb separation. Our measurements however reveal that it only takes ~ 25 times longer, corresponding to a more than 100-fold reduction.

## Anomalous relaxation time scaling induces long-ranged velocity correlations.

The anomalous relaxation time scaling makes a key prediction for the correlations of the absolute motion of DNA loci, quantified by the velocity cross-correlation $C_{\text{vv}}^{\left(\delta \right)}\left(t\right)=\langle v_{i}^{\left(\delta \right)}\left(t_{0}\right)\cdot v_{i}^{\left(\delta \right)}\left(t_{0}+t\right)\rangle t_{0}$. These correlations are determined by the relaxation time through the dimensionless ratio *δ/τ*, where *δ* is the experimental observation time-scale (Fig. 4g) [50]. Having determined the relaxation times *τ*, we can therefore make a parameter-free prediction of the correlations, which decay significantly more slowly than for the ideal Rouse model (Fig. 4h, green and grey lines). Notably, we find that the experimental correlations are quantitatively captured by this parameter-free prediction (Fig. 4h), including the full time-dependence of the correlations (Supplementary Section 6). This demonstrates that the anomalous relaxation time scaling indeed leads to long-ranged velocity cross-correlations of chromosomal loci.

## Discussion.

We developed an experimental approach to perform *in vivo* imaging of the joint dynamics of enhancer–promoter pairs with varying genomic separation and simultaneous monitoring of their transcriptional output. Observing the dynamics of pairs of DNA loci has only become possible recently and has been done for tagged DNA loci at a single fixed genomic distance [8, 30–32]. Here, we show how imaging across genomic separation allows us to measure the scaling exponents required to test polymer physics models proposed for chromosome organization and dynamics and their implications for transcription. The joint dynamics of pairs of DNA loci exhibit a combination of crumpled chain organization, fast subdiffusive dynamics, and a scale-dependent two-locus diffusivity. Together, these features lead to an anomalous scaling of relaxation times and long-ranged correlations in enhancer–promoter motion.

From a polymer physics perspective, our findings suggest that the relationship between static and dynamic properties in the generalized Rouse framework, which relies on the assumption of local friction, does not apply to chromosomes. They point to additional long-range interactions, such as hydrodynamic or active motor-mediated interactions [54, 55]. Indeed, the simplest hydrodynamic polymer model is the Zimm model [33]. It relaxes the assumption of local friction and includes long-range hydrodynamic interactions, and it predicts a scaling exponent of relaxation times with genomic distances *γ* = 1, close to our measured value of *γ* ≈ 0.8. Furthermore, the observed scale-dependent diffusivity points to additional heterogeneities along the polymer. They are necessary to capture chromosome dynamics, such as active processes [55], cross-linking [29], or the presence of condensates [18–20]. Together, these processes may orchestrate the anomalous scaling of relaxation times with genomic separation. In future work, the mechanistic underpinnings of our findings could be tested through polymer simulations [25, 29, 34–39, 52] and generate hypotheses for a new set of experiments.

The static and dynamic properties of the system determine the scaling of relaxation times, which in turn set the time-scale for distal enhancer–promoter encounters. We find a seemingly optimal combination of these exponents that yield relaxation times much less dependent on genomic separation than expected based on existing polymer models. This reduced dependence on distance implies that the probability of reaching and maintaining the spatial proximity required for transcription is similar for enhancers dispersed across the chromosome, allowing them to find their target promoter efficiently. Overall, our findings have crucial implications for the spatio-temporal organization of the cell nucleus, including the dynamics of long-range focal contacts [56] and mammalian enhancer-promoter interactions [9–12, 44].
